# Preliminary study of biliary microbiota and identification of bacterial species associated with pigmented gallstone formation

**DOI:** 10.3389/fcimb.2025.1532512

**Published:** 2025-03-20

**Authors:** Riyuan Zhang, Chong Chen, Suhua Zheng, Jianwei Zhang, Weiling Chen, Zhimin Chen

**Affiliations:** Department of Hepatobiliary and Pancreatic Surgery, Pingyang Hospital of Wenzhou Medical University, Wenzhou, Zhejiang, China

**Keywords:** pigment gallstones, β-glucuronidase, *Streptococcus*, parabacteroides merdae, 16S rRNA sequencing

## Abstract

**Background:**

Pigmented gallstone disease (PGS) is prevalent in China. Biliary microbiota is certified to be related to the PGS formation.

**Methods:**

We performed 16S sequencing on both bile and gallstone samples in 16 patients with gallstone disease. We analyzed the microbial composition of the biliary tract and identified crucial bacteria related to the formation of PGS.

**Results:**

Biliary tract bacterial composition analysis showed heterogeneity of dominated genus among individuals and correlation in bacterial composition between bile and gallstones. We screened 10 prevalent genera with significant abundance in the bile and gallstones. *Actinomyces*, *Streptococcus*, and *Achromobacter* had a significantly higher abundance in gallstones than in bile (*P* < 0.05). Furthermore, we identified 32 species that harbored *uidA*, *pldA*, and *plc* genes that encoded *β*-glucuronidase or phospholipase. Finally, we observed an enriched membrane transport for bile resistance through biliary microbiota.

**Conclusion:**

*β*-glucuronidase-producing *Streptococcus* spp., including *Streptococcus dysgalactiae* and *Streptococcus agalactiae*, and *Parabacteroides merdae*, harbored both *uidA* and *pldA* genes and were found to be crucial bacterial species in PGS formation.

## 1 Introduction

Gallstone disease is prevalent in China, especially pigmented gallstones (PGS). With age, the prevalence of gallstone disease reaches 16% and exceeds 20% in some regions (Su et al., 2020). Previous studies have linked biliary infection with gallstone development and indicated that bacteria may act as the nucleating factor initiating the formation of PGS (Stewart et al., 1987; Lee et al., 2010; Kose et al., 2018). Many bacteria, such as *Escherichia coli*, *Klebsiella pneumoniae*, *Enterococcus faecium*, *Enterobacter cloacae*, and *Pseudomonas aeruginosa*, have been identified in bile or gallstones through cultivation or polymerase chain reaction. Studies have shown a high prevalence of the phyla Proteobacteria, Firmicutes, Bacteroidetes, and Actinobacteria in the primary choledocholithiasis group (Chen et al., 2019).The microbiome is involved in the pathogenesis of cholelithiasis through several pathways (Dan et al., 2023). The bacterial products, *β*-glucuronidase and phospholipase, and bacterial biofilm are the key points. Helicobacter species induce gallstone formation by precipitating calcium. Therefore, an improved understanding of the biliary microbiota would be helpful for studies on bacteria-related gallstone pathogenesis and the formation of PGS. In this study, based on 16S sequencing, we reconstructed the composition of biliary microbiota, identified bacteria related to PGS formation, and planned to further establish the relationship between biliary microbiota and PGS formation.

## 2 Materials and methods

### 2.1 Patients and sample collection

The study conformed to the ethical guidelines of the 1975 Declaration of Helsinki, and all individuals provided written informed consent upon enrollment. This study was approved by the Medical Ethics Committee of Pingyang County People’s Hospital. Sixteen individuals (average age: 69.00 ± 10.32 years; 6 women, 10 men) who were diagnosed with cholangitis and choledocholithiasis using B-mode ultrasonography and computed tomography were enrolled. Individuals 1, 4, 5, 7, and 14 had a history of surgery on the digestive system, and individuals 10, 13, 15, and 16 had a history of surgery on the biliary system for more than 4 years. The participants underwent choledochoscopy, choledocholithotomy, and lithotripsy at this time and 5 mL bile samples and brown GBS samples were collected from the common bile ducts simultaneously. The procedure for sample collection was performed under strict sterile conditions. Samples were immediately placed in sterile centrifuge tubes separately and stored at -80°C for subsequent study.

### 2.2 DNA extraction and purification

The surface of the gallstone was washed with phosphate-buffered saline. Each gallstone was cut from the center using a sterile blade and scraped to obtain the inner matrix. After grinding and drying to a constant weight, 200 mg of gallstone powder was incubated with 1 mL of 1% sodium lauryl sulfate at room temperature overnight. Lithium chloride (7 mol/L) was added to give a final concentration of 1.5 mol/L. The mixture was shaken for 1 minute, centrifuged at 12,000 g for 15 minutes, and the supernatant separated. The DNA was then extracted twice with an equal volume of saturated phenol and once with an equal volume of chloroform-isoamyl alcohol. A QIAamp DNA Mini kit (QIAGEN, Germany) was used for further purification.

A 500 µL volume of bile was centrifuged at 12,000 g for 10 minutes and the filtrate was collected. Added to the filtrate was an equal volume of lysis buffer [10 mM Tris-Hcl (pH = 8.5), 10 mM EDTA, 100 mM NaCl, 0.5% sodium lauryl sulfate) and 50 µL of proteinase K and this was incubated at 55°C for 8 hours. A QIAamp DNA Mini kit (QIAGEN, Germany) was used for further purification.

### 2.3 16S rRNA amplicon sequencing

A two-step procedure for 16S rRNA library construction was used (Shen et al., 2015). The V3-V4 regions of the 16S rRNA gene cover a longer segment of the 16S rRNA, including two hypervariable regions (V3 and V4), which leads to improved bacterial classification resolution. The V3-V4 regions of the bacterial 16S rRNA gene were amplified using universal primer pairs 341F (5’- ACTCCTACGGGAGGCAGCA-3’) and 805R (5’-GACTACHVGGGTATCTAATCC-3’). Sterile water was used as a negative control during the whole amplification and library preparation process. The 16S rRNA libraries were sequenced on the Illumina NovaSeq PE250 platform (Illumina, USA) to generate 2 × 250-bp paired-end reads.

### 2.4 Pre-processing of off-machine data

The Qiime 2 analysis process was adopted and Divisive Amplicon Denoising Algorithm 2 (DADA2) was used to denoise the raw data, which was equivalent to clustering with 100% similarity. Furthermore, we used Qiime 2’s built-in DADA2 for low-quality filtering operations such as adapters, primer removal, and denoising by executing truncQ=2, F end maxEE=5, and R end maxEE=2. Finally, we used DADA2 to merge the double-ended sequences and de-chimerism to obtain clean reads.

### 2.5 Analysis of 16S sequencing data

Sequence data analysis mainly used Qiime 2 (Qiime 2-2020.2) and R (4.0.2). The denoised sequences were directly made de-redundant to obtain the feature information using DADA2. According to the silva-132-99 database and the primer pairs of the V3-V4 regions, Qiime2 was used to trim the reference genome’s, Silva132, 99% features to the V3-V4 region and train the classifier. The trained naive Bayes classifier was used to classify the features to obtain the corresponding species classification information. Each feature was defined as an amplicon sequence variant (ASV). Qiime 2 was used to calculate the alpha diversity index (Chao1, ACE, Shannon’s, and Simpson’s indices). R was used to perform the alpha diversity analysis between groups based on the Wilcoxon test (R phyloseq package). Principal coordinates analysis (PCoA) was conducted based on the Bray–Curtis distance (phyloseq/vegan package). ANOSIM analysis (R phyloseq/vegan package), Pearson’s correlation analysis, and analysis of differential bacteria based on the Wald test (R DESeq2 package) were conducted between groups. We used PICRUSt2 (https://github.com/picrust/picrust2) to predict the metabolic function of the flora by searching the KEGG GENOME Database and KEGG PATHWAY Database. *P* < 0.05 or adjusted *P* < 0.05 was considered to be statistically different.

## 3 Results

### 3.1 The sequencing data

Bile samples of individuals 9 and 14 failed to produce nucleic acid extract. We performed high throughput sequencing of the 16*S rRNA* V3-V4 regions of bacteria in 14 bile samples (group A) and 16 gallstone samples (group B), obtaining average raw reads of 177,548.50 ± 37,669.28 in group A and 147,736.69 ± 58,253.30 in group B. After multiple filtrations, the number of final clean reads was 144,080.70 ± 35,753.81 in group A and 131,720.90 ± 51,808.84 in group B. Furthermore, we performed species classification annotations using feature information generated by DADA2, with 43 to 1,142 ASVs obtained in group A and 43 to 1,392 ASVs obtained in group B. The number of ASVs among 16 samples in the group was quite different. After filtering out low-abundance ASVs (<10), a total of 813 ASVs were shared by the two groups, and the number of unique ASVs in group A and in group B was 1,615 and 1,142, respectively.

### 3.2 Diversity analysis

**Figure 1:**
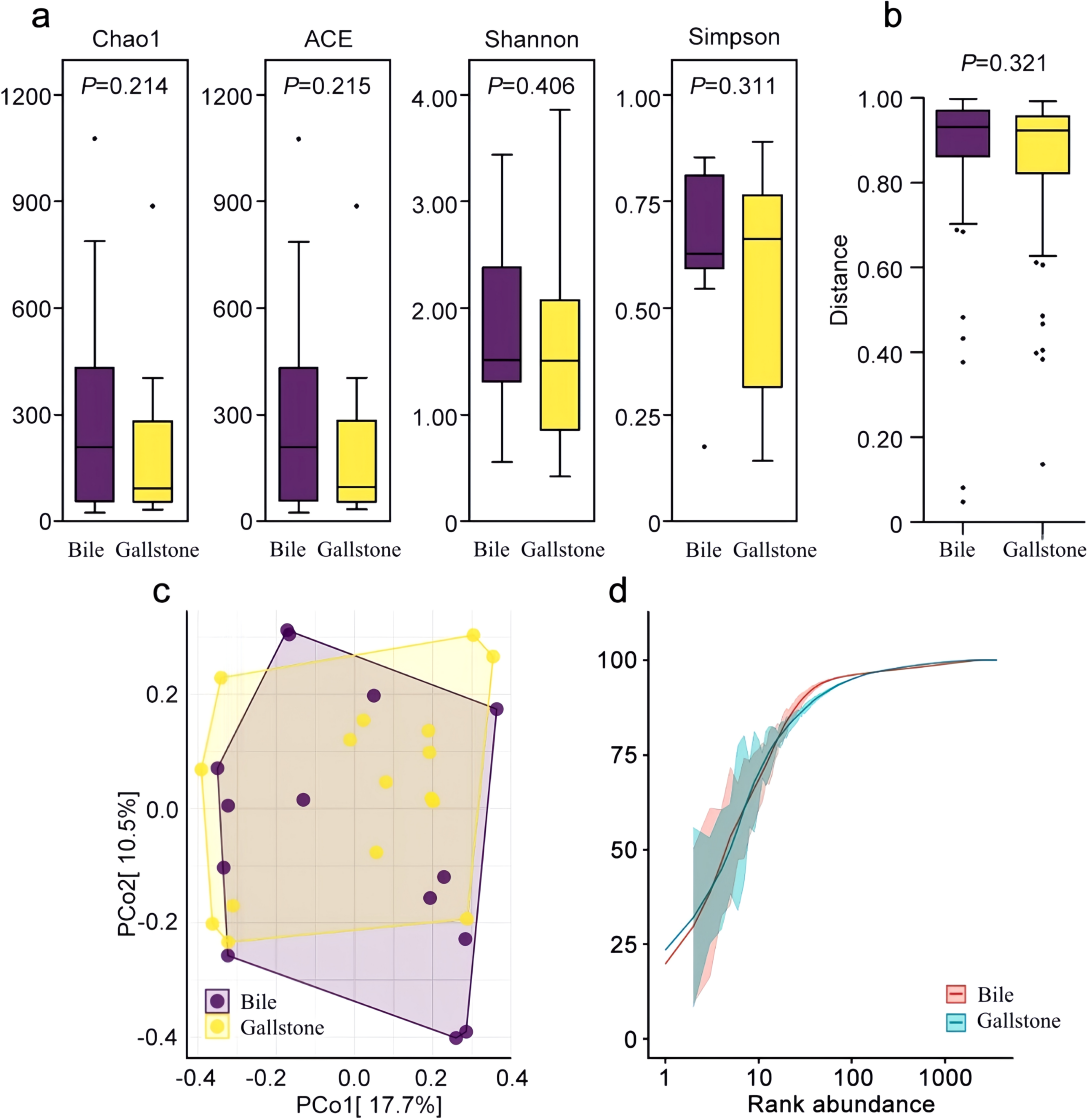
Microbial community characteristics of the bile and gallstone samples. **(a)** Four indexes of alpha diversity. Purple and yellow boxplots denote distributions of 16S sequencing of bile samples and gallstone samples, respectively. **(b)** Beta diversities based on the Wilcoxon rank sum test. **(c)** Beta diversities are presented as a PCoA matrix diagram. **(d)** Rare faction curves of the two groups.

Diversity differences in bile microbiota and gallstone microbiota were observed. Analysis using the Chao1, ACE, Shannon’s, and Simpson’s indices indicated that the gallstone microbiota had a reduced bacterial diversity compared with the bile microbiota, without a statistical difference (*P* > 0.05) (**Figure 1a**). Wilcoxon rank sum test analysis based on the Bray–Curtis algorithm found that there was no statistical difference in the beta diversities index between the two groups (*P* = 0.321) (**Figure 1b**). PCoA indicated a similar microbial composition (**Figure 1c**). Rare faction curves showed the abundance and uniformity of the two groups (**Figure 1d**).

### 3.3 Bacterial composition of biliary tract

Generally speaking, according to the abundance ranking of ASVs, Proteobacteria (14.78%), Firmicutes (9.66%), Bacteroidota (2.54%), Synergistota (1.97%), Actinobacteriota (0.51%), Fusobacteriota (0.17%), and Campilobacterota (0.09%) were the top seven phyla detected in the biliary tract samples. At the genus level, *Escherichia Shigella* (9.90%), *Enterococcus* (5.35%), *Bacteroides* (2.05%), *Pseudomonas* (1.97%), *Clostridium sensu stricto* 13 (1.79%), *Pyramidobacter* (1.72%) and a genus of Enterobacteriaceae (1.59%) were the top seven genera detected in the biliary tract samples. In this study, a total of 259 bacterial species were identified. *Bacteroides fragilis* (2.00%), *Pyramidobacter piscolens* (1.97%), *Streptococcus dysgalactiae* (0.36%), *Streptococcus anginosus* (0.15%), *Campylobacter concisus* (0.07%), *Lactobacillus kefiranofaciens* (0.07%), *Actinomyces gerencseriae* (0.05%), *Vibrio fluvialis* (0.05%), *Bifidobacterium animalis* (0.04%), *Helicobacter typhlonius* (0.01%), and *Bacteroides sartorii* (0.01%) had a relative abundance of more than 0.01%.

**Figure 2:**
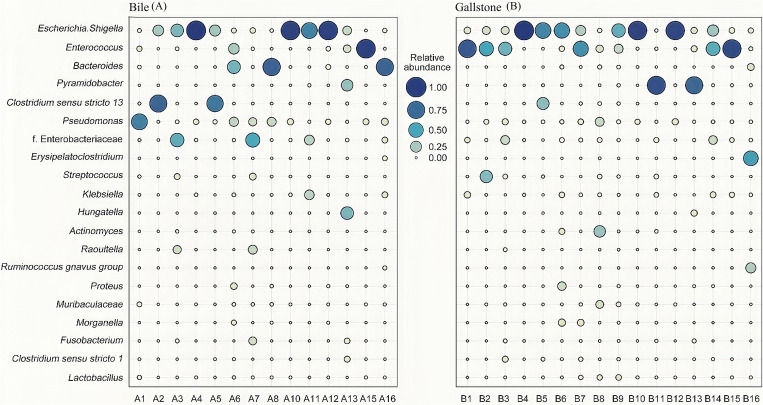
Microbial community characteristics generated by 16S sequencing. **(A, B)** Relative abundances of top 10 genera in the bile and gallstone samples. Lack of A9 and A14 due to failure of nucleic acid extraction. The genera names for **(A, B)** are shown on the y-axis of **(A)**. f. Enterobacteriaceae represented an undefined genus of Enterobacteriaceae.

Biliary tract bacterial composition analysis showed heterogeneity among individuals. Individuals 4, 10, and 12 were dominated by *Escherichia Shigella* in both their bile and gallstone samples (over 90%), and followed by 3% to 5% of *Pseudomonas*. *Enterococcus* dominated in the bile and gallstone samples of individual 15, and only in the gallstone sample of individual 1, with a proportion of 90% approximately. Individual 13 had a large proportion of *Pyramidobacter* (bile sample, 29.19%; gallstone sample, 77.85%) and *Hungatella* (bile sample, 36.54%; gallstone sample, 5.39%). *Pyramidobacter* only dominated in the gallstone sample of individual 11 (88.01%) and not in their bile sample (0.31%). Individual 5 was dominated by *Escherichia ShigellaI* (bile sample, 25.70%; gallstone sample, 63.86%) and *Clostridium sensu stricto* 13 (bile sample, 67.59%; gallstone sample, 33.51%), and individual 2 had the same dominating bacteria in their bile sample (*Escherichia ShigellaI*, 23.65%; *Clostridium sensu stricto* 13, 75.51%) but not in their gallstone sample (*Escherichia ShigellaI*, 11.06%; *Clostridium sensu stricto* 13, 0.00%). Individuals 6, 8, and 16 had a relative abundance of *Bacteroides* (6, 39.24%; 8, 77.76%; 16, 76.13%) and *Pseudomonas* (6, 16.00%; 8, 15.03%; 16, 7.32%) in their bile samples, however, the bacteria were significantly different in their gallstone samples, dominated by *Escherichia Shigella* (6, 59.98%), *Actinomyces* (8, 30.00%), and *Erysipelatoclostridium* (16, 53.92%), respectively (**Figure 2**).

We did not observe a correlation between biliary microbiota and a history of previous surgery. The ANOSIM analysis showed that the difference in biliary microbial composition between the groups was slightly greater than the difference within the groups, without a statistical difference (*R* = 0.026, *P* = 0.227). Bacterial microbiota in the bile and gallstones within individuals exhibited a high correlation (*P* < 0.001 for all), except for individuals 1 (*r* = 0.050, *P* = 0.098), 2 (*r* = 0.051, *P* = 0.090), 7 (*r* = 0.055, *P* = 0.071), and 11 (*r* = 0.022, *P* = 0.475). In general, the microbial composition of bile and gallstones was the same. Thus, differential bacteria may be a key factor in PGS formation.

### 3.4 Differential bacteria between groups

Although most genera that occurred in bile were also detected in gallstones, the abundance and prevalence of these bacteria differed greatly. Based on the Wald test, a total of 128 genera showed a statistical difference in abundance between bile and gallstones (*P* < 0.05). Twenty-nine genera were observed to be more abundant in gallstones than in bile.

**Figure 3:**
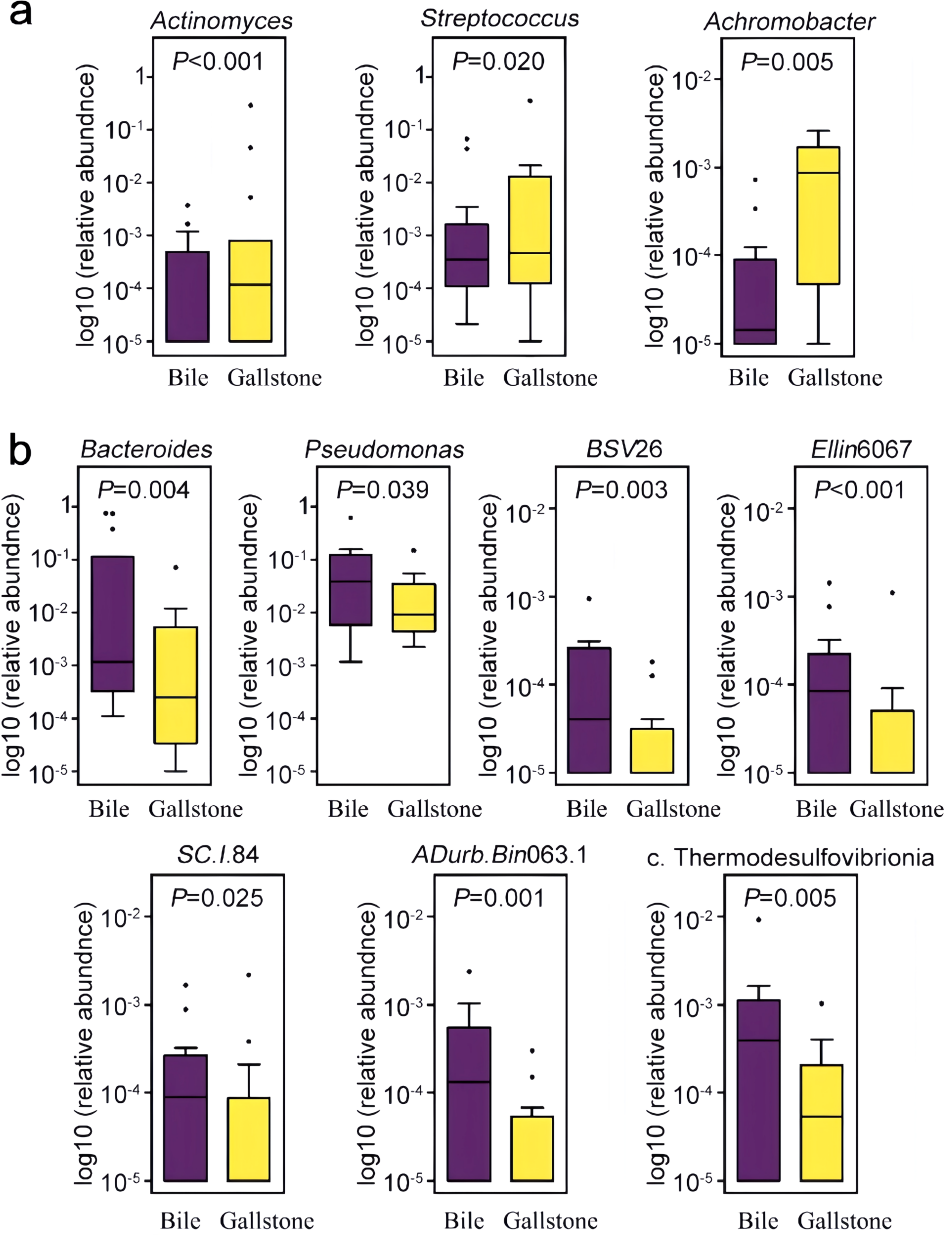
Distribution of differentially abundant genera in the bile and gallstone samples. Genera that were statistically increased **(a)** and statistically decreased **(b)** in relative abundance in the gallstone samples compared to bile samples are illustrated. Genera were filtered by the criterion of detection in at least 50% (15/30) of the samples. *BSV*26, a member of the Kryptoniales order; *Ellin*6067, a member of the *Nitrosomonadaceae* family; *SC*.*I*.84, a member of the Burkholderiales order; *ADurb*.*Bin*063.1, a member of the *Pedosphaeraceae* family; c. Thermodesulfovibrionia, an uncultured genera of the Thermodesulfovibrionia class.

Among them, 10 prevalent genera identified in at least 15/30 samples were selected, with a 4.23 to 29.19-fold difference between groups (**Figure 3**). Three genera (*Actinomyces*, *Streptococcus*, and *Achromobacter*) had a significantly higher abundance in gallstones than in bile. With respect to these 10 genera prevalent among individuals, *ADurb*.*Bin*063.1 was detected in 10 bile samples and in only 5 gallstone samples, with a statistical difference (*χ* ^2^ = 4.821, *P* = 0.028). The prevalence of the other nine genera was not statistically different (*P* > 0.05).

### 3.5 Species related to PGS formation

**Figure 4:**
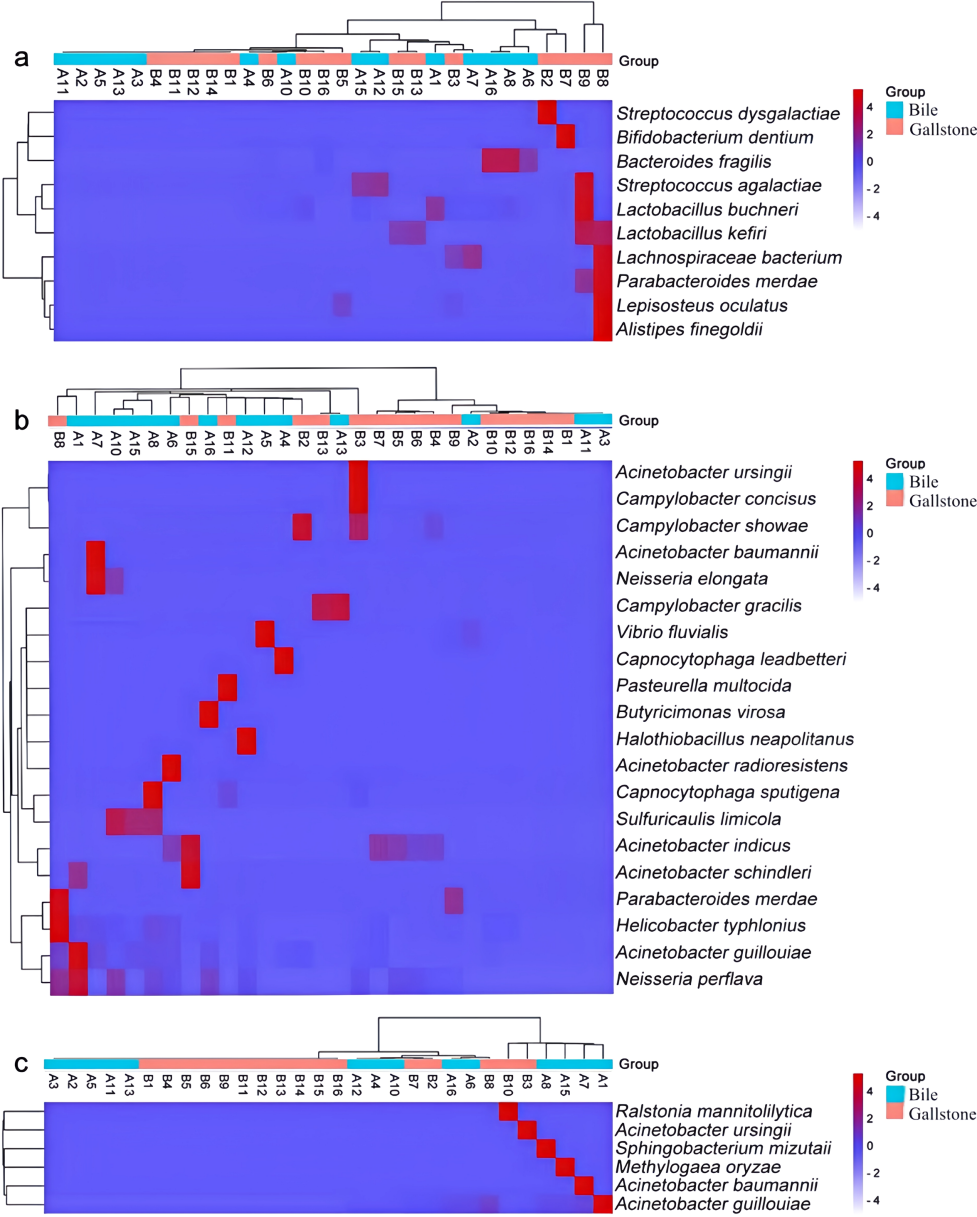
Heatmap with the crucial bacterial species harboring the *uid*A **(a)**, *plc*A **(b)**, and *plc* **(c)** genes. The heatmap color scale quantifies the log_10_ average relative abundance of species in the biliary tract, from purple (none or low abundance) to red (high abundance). The dendrograms illustrate the relationship between samples and groups, clustered by the Wald test.

We further investigated genes that might be related to PGS formation. Previous studies have demonstrated potential associations between PGS formation and bacterial products *β*-glucuronidase and phospholipase. Among the 259 identified biliary species, we extracted 32 species harboring genes encoding these enzymes by searching the KEGG GENOME database. Thus, 10 species were predicted to have the *uidA* gene encoding *β*-glucuronidase. All of the 10 species were detected in bile, and only 5 of the 10 species were detected in gallstones. The *pldA* gene was more abundant in bacteria in bile. In total, 20 species harbored the *pldA* gene encoding phospholipase A1/A2, with 12 of 20 species detected in bile and 15 of 20 species detected in gallstones. *Parabacteroides merdae* possessed both the *uidA* and *pldA* genes. Six species harbored the *plc* gene encoding phospholipase C (**Figure 4**).

### 3.6 Inferred metabolic function of biliary tract flora

**Figure 5:**
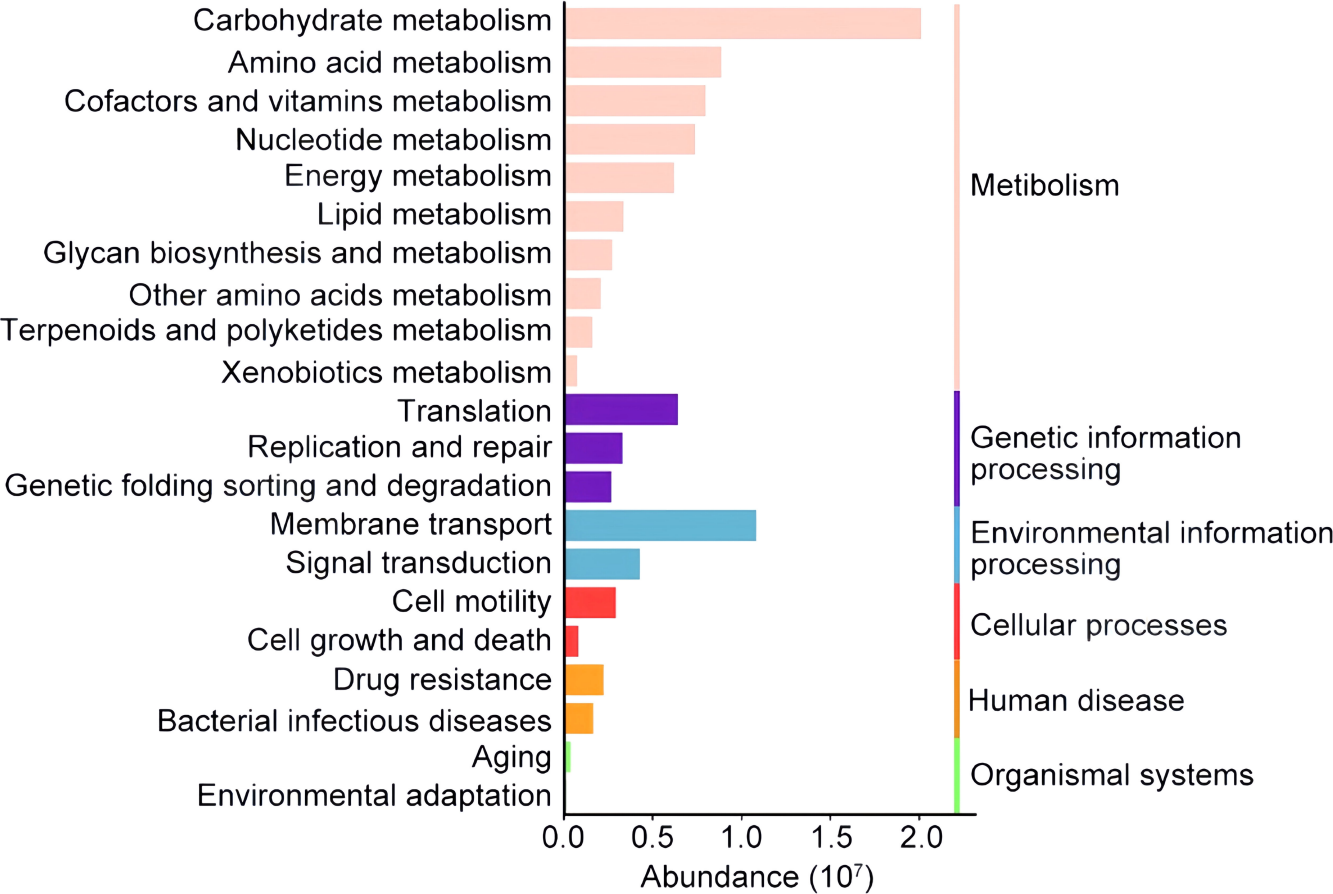
Histogram of the major functional pathways identified in the biliary tract of patients with PGS. The colored column showed the relative abundance of the metabolic pathway categories of microbiota in the biliary tract. The metabolic pathways are clustered according to the KEGG PATHWAY database on the right. Only the top 21 metabolic pathways are shown here.

Additionally, we used PICRUSt2 to predict the metabolic function of the biliary tract flora and clustered them based on the KEGG PATHWAY database. Metabolism was the basic life activity of biliary microbiota. There was an overall higher abundance of genes involved in carbohydrate metabolism than in amino acid metabolism, consistent with the report of Kose et al. (2018). Enriched membrane transport pathways maintain the exchange of substances between the bacterial organism and biliary environment, and were predicted to probably promote bile resistance (**Figure 5**).

## 4 Discussion

Studies have identified bacteria in the majority of patients with PGS and conclude that bacteria play an important structural and functional role in the formation of PGS (Stewart et al., 1987; Wu et al., 2013; Shen et al., 2015). Bacteria detected in bile and gallstones frequently occur in the digestive tract. Ye et al. (2016) compared bacterial communities from the biliary tract and upper digestive tract of patients with gallstones and found that the biliary bacteria probably originated from a retrograde infection of gut bacteria. We found that all of the top seven genera in the biliary tract were intestinal microbiota, which explains the connection between biliary tract flora and intestinal flora (Kamada et al., 2013; Lin et al., 2018). Although bacteria come from the digestive tract, biliary microbiota have unique flora characteristics.

The dominant microbiota in the gut and intestines are abundant and uniform (Ruan et al., 2017; Lin et al., 2018). Different from them, the dominant biliary microbiota is relatively single and commonly dominated by several genera, generally one to three genera. Studies have reported that *Escherichia Shigella* and *Enterococcus* are most frequent whether detected by bile culture or high-throughput sequencing (Kaufman et al., 1989; Dyrhovden et al., 2020). The dominance was only found in the gallstone samples (12/16), but not in the bile samples (6/14) in this study. A consensus has not yet been reached on which type of microbiota is common to PGS. We found that *Pyramidobacter*, *Hungatella*, *Clostridium sensu stricto* 13, *Bacteroides*, and *Pseudomonas* comprise a relative proportion of genera in biliary tract in some individuals, which revealed that the microbial composition of the biliary tract exhibited heterogeneity (Wu et al., 2013; Ye et al., 2016). We did not observe a correlation between biliary tract flora and a history of previous surgery. Thus, the heterogeneity implies that the biliary microbiota may be shaped by more complicated factors, such as diet, lifestyle, or host immune responses. The possibility of accidental deviation in the reflux of biliary microbiota from the gut or duodenum cannot be ruled out. This difference may also be due to altered bile microbiota following stone nucleation. The microbiota in gallstones behaves like the bile microbiota ecology at a certain stage of stone formation. However, we found that the microbial composition of bile and gallstones was correlated at the time of detection, so the changes in bile during gallstone formation may not be as dramatic as previously thought in most individuals. It is possible that some bacteria were accidentally encapsulated in the stone during gallstone formation, resulting in the heterogeneity of the dominant genera.

There is no doubt that the microorganisms of gallstones are derived from bile. When microorganisms metabolize substances in bile, such as bilirubin, it promotes the deposition of bilirubin in bile. Nearby bacteria involved in bilirubin deposition may be adsorbed on the bilirubin crystals and then deposited together to form gallstones. Therefore, exploring the differential bacteria between gallstones and bile can clarify the microorganisms that are active during gallstone formation and those that are more associated with PGS formation. We confirmed a significant change in biliary microbiota between bile and gallstones. The prevalence of the genera *Actinomyces*, *Streptococcus*, and *Achromobacter* was observed to be more abundant in gallstones than in bile. *Actinomyces* infection can cause an exceedingly rare actinomycosis characterized by a tendency to form cholecystitis and abdominal abscesses (Vyas et al., 2007). Potti et al. (1999) reported a case of gallbladder carcinoma associated with biliary actinomycosis. Studies (Frey and Freter, 1968; Miquel et al., 1998) have shown that cholecystitis is not critical for the formation of gallstones, so it is speculated that the formation of gallstones and excessive overgrowth of *Actinomyces* may actually be the causes of cholecystitis or cholangitis. *Achromobacter*, a pigment-producing species, was detected in the bile of patients with benign and malignant pancreaticobiliary diseases (Poudel et al., 2023). *Achromobacter* catalyze the oxidation of L- tryptophan, yielding a red pigment, and probably play a key role in the color staining of brown gallstones (Krishnamurthi et al., 1969). Hancke and Marklein (1983) detected *β*-glucuronidase-producing group G *Streptococcus* in the bile, gallbladder wall, and livers of mongrel dogs with bile pigment gallstones. Previous studies also demonstrated that *β*-glucuronidase, a metabolite of *Streptococcus*, plays an important role in PGS formation (Wang et al., 2018). The deconjugation of *β*-glucuronidase can precipitate calcium bilirubinate which is conjugated with anionic glycoprotein, and cause the agglomeration of calcium bilirubinate crystals into stones (Maki, 1966). Thus, the overgrowth of *Streptococcus*, including *Streptococcus dysgalactiae* and *Streptococcus agalactiae*, may contribute to the gallstone formation in this study.

*β*-glucuronidase and phospholipase accelerate the precipitation of calcium bilirubinate. Some other *β*-glucuronidase and phospholipase-producing bacteria were detected in biliary tracts. *β*-glucuronidase induces the hydrolysis of bilirubin diglucuronides to produce unconjugated bilirubin, resulting in the precipitation of calcium bilirubinate (Osnes et al., 1997). Phospholipase-producing bacteria were abundant in the bile samples, contributing to the PGS formation. Studies prove that brown PGS contains calcium palmitate (Sharma et al., 2015). Bacterial phospholipase on biliary phosphatidylcholine can result in the release of palmitic acid, which then combines with ionized calcium to form calcium palmitate. The precipitation of calcium palmitate produces solids that may be incorporated into the stones. Nearly one-third of the cultured strains of cholesterol gallstones secrete *β*-glucuronidase and phospholipase (Hu et al., 2022). *Parabacteroides merdae* harbored both *uidA* and *pldA* genes, encoded these two enzymes, and played a crucial role in PGS formation. In addition, *Parabacteroides merdae* is involved in bile acid metabolism to produce isallo-lithocholic acid. This derivative exerts a potent antibacterial effect against gram-positive multidrug-resistant pathogens in bile (Stewart et al., 1987), which may cause the enrichment of gram-negative bacteria in PGS (Kose et al., 2018). Extracellular polymeric substances produced by these gram-negative bacteria exhibit anionic properties that enable calcium and magnesium ions to bind with polymer strands, providing a tightly bound biofilm architecture outside stones (Donlan, 2002).

Biliary microbiota are known to survive in a variety of extreme environments, including those containing sodium and potassium salts, bile acids, cholesterol, phospholipids, commonly used antibiotic drugs, and heavy metals. A variety of genes are involved in bile resistance to protect bacteria from the toxic agents in bile, such as multidrug export efflux pumps, DNA and cell wall repair proteins (Kose et al., 2018). In this study, we observed an enhanced membrane transportation that can transfer harmful substances outside bacteria, avoiding burden and damage. At the same time, pathways such as DNA repair were also highly abundant in biliary microbiota.

While our research has yielded several significant findings, it is essential to acknowledge certain limitations that warrant attention in future studies. Notably, the PCoA revealed that the gallstone and bile samples were not distinctly separated. This observation indicates that there may be factors not fully accounted for that could have influenced the differences between these samples. For example, the gallstone samples may have been contaminated by bile, or vice versa, leading to potential overlap that obscures the distinctions between the two sample types. Additionally, the similarity in microbial communities or chemical compositions between the gallstone and bile samples may have further contributed to their overlap in the PCoA plot. The next step will involve investigating whether this overlap arises from intra-patient variability or if it necessitates the development of visualizations for paired data. Moreover, the relatively small sample size of this investigation may not adequately represent the broader population of patients with gallstones. This limitation could result in biased outcomes and may overlook differences among various demographic groups. Furthermore, a small sample size may reduce statistical power, increasing the likelihood of undetected important biological effects. To validate and expand upon our findings, future research should pursue several key directions: conducting multi-center, large-sample studies to enhance the representativeness and reliability of the results.

In summary, we initially obtained the microbial community of the bile and gallstone samples and identified species that may play a role in the formation of PGS. β-glucuronidase-producing *Streptococcus* was enriched in gallstones and correlated with PGS formation. Furthermore, we identified 32 crucial species that harbored *uidA*, *pldA*, or *plc* genes encoding β-glucuronidase or phospholipase colonization in the biliary tract. The identification of PGS formation-related bacterial species provides a foundation for further research on the formation and treatment of PGS.

## Data Availability

The data presented in our study have been deposited in the NCBI repository under the accession number PRJNA1236259.
